# *N*-Heterocyclic carbene–palladium catalysts for the direct arylation of pyrrole derivatives with aryl chlorides

**DOI:** 10.3762/bjoc.9.35

**Published:** 2013-02-12

**Authors:** Ismail Özdemir, Nevin Gürbüz, Nazan Kaloğlu, Öznur Doğan, Murat Kaloğlu, Christian Bruneau, Henri Doucet

**Affiliations:** 1 Chemistry Department, Faculty Science and Arts, İnönü University, 44280 Malatya, Türkiye; 2 Institut Sciences Chimiques de Rennes, UMR 6226 CNRS-Université de Rennes "Organometalliques, Material and Catalysis", Campus de Beaulieu, 35042 Rennes, France. Fax: +33 (0)2-23-23-69-39; Tel: +33 (0)2-23-23-63-84

**Keywords:** aryl chlorides, atom-economy, C–H bond activation, C–H functionalization, carbenes, palladium, pyrroles

## Abstract

New Pd–NHC complexes have been synthesized and employed for palladium-catalyzed direct arylation of pyrrole derivatives by using electron-deficient aryl chlorides as coupling partners. The desired coupling products were obtained in moderate to good yields by using 1 mol % of these air-stable palladium complexes. This is an advantage compared to the procedures employing air-sensitive phosphines, which have been previously shown to promote the coupling of aryl chlorides with heteroarenes.

## Introduction

*N*-Heterocyclic carbenes (NHC) have emerged as an important class of ligands in the development of homogeneous catalysis [1–9]. Such ligands, which are electronically and sterically tunable, and which generally form thermally stable compounds with different metal ions, are strong σ-donors. These qualities have rendered N-heterocyclic carbene ligands as classical substitutes to phosphines in organometallic catalysis [10–14]. This is especially true for palladium-catalyzed coupling reactions. Pd–NHC catalysts [15] have proven to be excellent alternatives to catalytic systems involving palladium associated to tertiary phosphine ligands [16–19].

**Figure 1:**
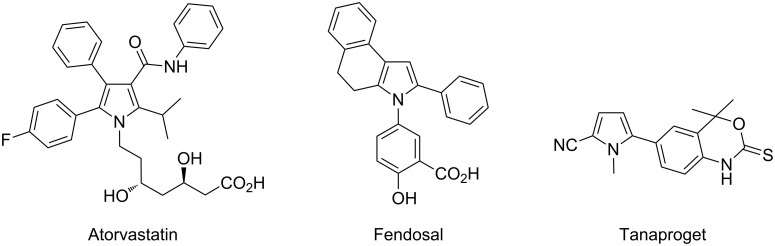
Examples of pyrrole-containing bioactive compounds.

The introduction of aryl groups at C2 or C5 positions of pyrroles is an important research area in organic synthesis as such motives are known to be present in several bioactive molecules, such as Atorvastatin, which is used for lowering blood cholesterol, Fendosal, which is an anti-inflammatory agent, or Tanaproget, which is a progesterone-receptor agonist (Figure 1).

The palladium-catalyzed direct arylation of various heteroaromatics including pyrroles by a C–H bond activation using aryl halides has met great success in recent years, allowing the synthesis of a wide variety of arylated heteroaromatics in only one step [20–25]. However, there are still limitations for these reactions in terms of aryl halide or heteroaromatic tolerance. Up to now, very few examples of palladium-catalyzed direct arylations of pyrroles by using aryl chlorides have been reported, [26–27]. Daugulis and co-workers recently described the arylation of pyrrole derivatives with a variety of aryl chlorides using 5 mol % of Pd(OAc)_2_ associated to 10 mol % of Cy_2_P-*o*-biphenyl as the catalyst [26]. However, in most cases, such couplings were performed with aryl bromides or iodides [28–39].

The influence of mono- or diphosphines as ligands for the palladium-catalyzed coupling of heteroarenes with aryl halides through a C–H bond activation has been largely explored. On the other hand, the influence of carbene ligands for such couplings remains largely unexplored [40–47]. Quite congested *N*-heterocyclic carbene–palladium catalysts have been employed by Fagnou and co-workers to promote intramolecular direct arylations of arenes [40]. A few examples of couplings of aryl bromides and iodides employing Pd–NHC complexes have also been reported [41–45]. For example, Sames and co-workers described the use of imidazolylidene carbene ligands for the Pd-catalyzed direct arylation of pyrroles or indoles using bromobenzene and aryl iodides [42]. They observed that an important steric demand on the carbene ligand led to better results. Recently, the use of palladium(II) acetate complexes bearing both a phosphine and a carbene ligand, was reported by Lee and co-workers for the direct arylation of imidazoles with some aryl chlorides [46]. However, to our knowledge, *N*-heterocyclic carbene ligands have not yet been employed for the palladium-catalyzed direct arylation of pyrroles with aryl chlorides. As carbene ligands have proved to be very useful for several palladium-catalyzed reactions involving aryl chlorides, we decided to explore their potential for the direct 2- or 5-arylation of pyrrole derivatives.

## Results and Discussion

**Scheme 1:**
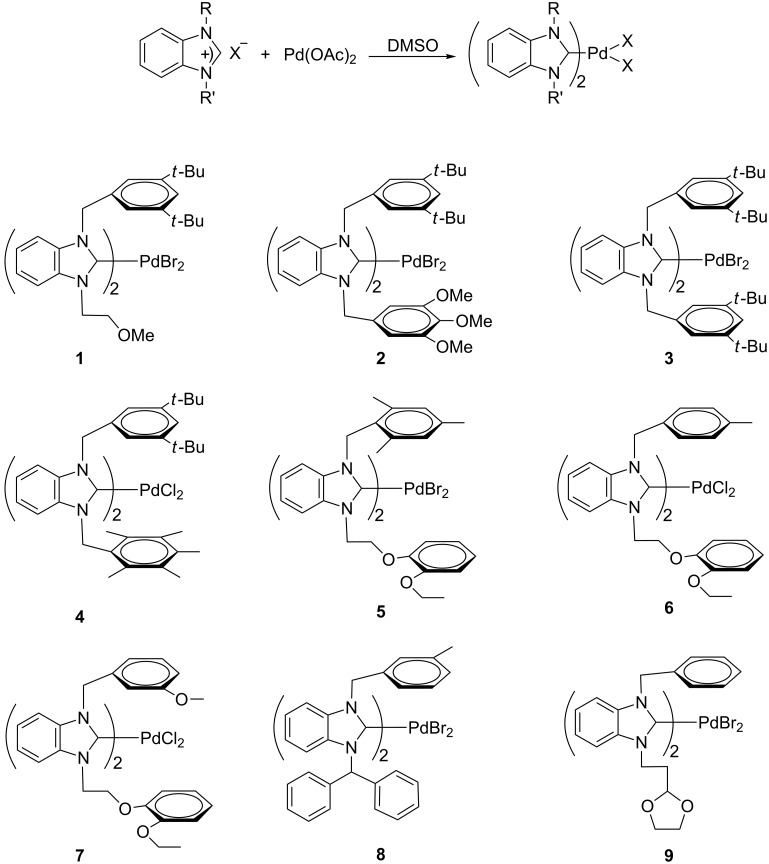
Synthesis of Pd–NHC complexes.

First, a range or Pd–NHC complexes employing a variety of carbene ligands was prepared (Scheme 1). The deviations from the accustomed structures of palladium–NHC complexes can be attributed to steric rather than to electronic factors [48]. The use of quite congested carbene ligands has been found to be required for the palladium-catalyzed direct arylation of pyrroles, indoles, benzothiophene [42,45] or arenes [40]. Therefore, we employed carbenes bearing relatively bulky N-substituents. The reaction of Pd(OAc)_2_ with the corresponding benzimidazolium halides in DMSO at 60–110 °C gave **1**–**9** in 53–87% yields (Scheme 1). The geometry of these complexes was not defined, as no crystals suitable for X-ray analysis could be obtained.

### Arylation with Pd–NHC complexes

We initially examined the direct 5-arylation of 1-methylpyrrole-2-carboxaldehyde (**10**) with 4-chlorobenzonitrile (**11**) using these nine Pd–NHC complexes. We had previously observed that with this pyrrole derivative a high yield of 89% could be obtained in the presence of only 0.5 mol % of a triphosphine associated to Pd(OAc)_2_ as the catalyst [27]. With complexes **2**, **3**, **8** and **9**, a high conversion of 4-chlorobenzonitrile (**11**) and good yields of the coupling product **16** were obtained (Table 1, entries 1–9). Then, in order to confirm this trend, 2-chlorobenzonitrile (**12**) and 4-(trifluoromethyl)chlorobenzene (**13**) were reacted with 1-methylpyrrole-2-carboxaldehyde (**10**) by using this library of complexes (Table 1, entries 10–27). Again, complexes **2**, **8** and **9** were found to be effective catalysts for this transformation, and led to a high conversion of 2-chlorobenzonitrile (**12**) to give **17** in 55–60% yield (Table 1, entries 10–18). For 4-(trifluoromethyl)chlorobenzene (**13**), the best results were obtained with catalysts **2** and **8** to give **18** in 76% and 74% yields, respectively (Table 1, entries 20 and 26). Then, the reactivity of 4-chlorobenzaldehyde (**14**) and 4-chloroacetophenone (**15**) was examined by using complexes **2**, **8** and **9**. For both substrates the best yields of products **19** and **20** of 41% and 50% were obtained with complex **8** (Table 1, entries 28–33).

**Table 1:** Direct arylation of 1-methylpyrrole-2-carboxaldehyde (**10**) with chlorobenzene derivatives.^a^

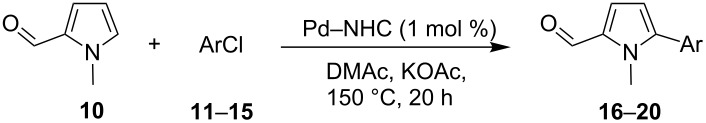					

Entry	ArCl	Pd–NHC	Product	Conv. (%)^b^	Yield (%)^b^

1 2 3 4 5 6 7 8 9	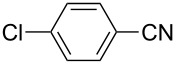 **11**	**1** **2** **3** **4** **5** **6** **7** **8** **9**	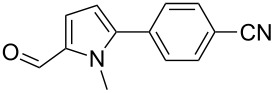 **16**	68 99 99 69 64 46 45 80 74	57 **87** **83** 61 57 36 37 **72** **65**

10 11 12 13 14 15 16 17 18	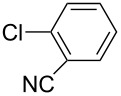 **12**	**1** **2** **3** **4** **5** **6** **7** **8** **9**	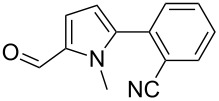 **17**	63 78 38 45 32 32 26 65 67	23 **60** 11 33 7 5 20 **58** **55**

19 20 21 22 23 24 25 26 27	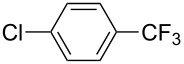 **13**	**1** **2** **3** **4** **5** **6** **7** **8** **9**	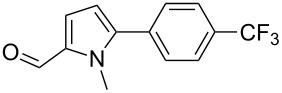 **18**	100 98 100 100 86 77 100 98 98	56 **76** 34 32 8 40 28 **74** 21

28 29 30	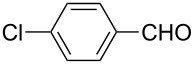 **14**	**2** **8** **9**	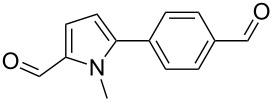 **19**	11 70 74	2 **41** 38

31 32 33	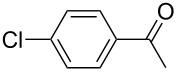 **15**	**2** **8** **9**	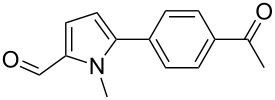 **20**	24 73 74	9 **50** 47

^a^Reaction conditions: Pd–NHC (0.01 mmol), aryl chloride (1 mmol), 1-methylpyrrole-2-carboxaldehyde (**10**, 2 mmol), KOAc (2 mmol), DMAc (3 mL), 20 h, 150 °C. ^b^Determined by GC and NMR.

The reactivity of 2-acetyl-1-methylpyrrole (**21**) was similar to 1-methylpyrrole-2-carboxaldehyde (**10**, Table 2). Complexes **8** and **9** promoted an almost complete conversion of 2- and 4-chlorobenzonitrile, and of 4-(trifluoromethyl)chlorobenzene to give the desired coupling products **22**–**24** in good yields. On the other hand, low to moderate yields were obtained with complexes **1**, **4** and **6**.

**Table 2:** Direct arylation of 2-acetyl-1-methylpyrrole (**21**) with chlorobenzene derivatives.^a^

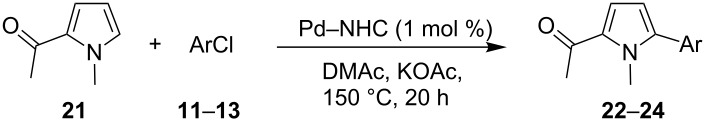					

Entry	ArCl	Pd–NHC	Product	Conv. (%)^b^	Yield (%)^b^

1 2 3 4 5 6	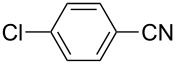 **11**	**1** **4** **6** **7** **8** **9**	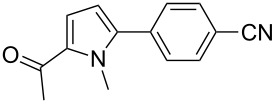 **22**	58 64 79 77 98 99	54 61 45 71 **85** **85**

7 8 9 10	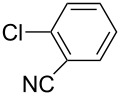 **12**	**1** **4** **8** **9**	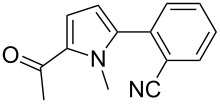 **23**	62 71 94 90	38 59 **78** **78**

11 12 13 14	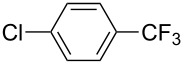 **13**	**1** **4** **8** **9**	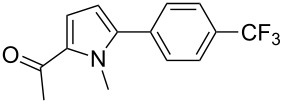 **24**	79 80 92 95	21 42 **77** **76**

^a^Reaction conditions: Pd–NHC (0.01 mmol), aryl chloride (1 mmol), 2-acetyl-1-methylpyrrole (2 mmol), KOAc (2 mmol), DMAc (3 mL), 20 h, 150 °C. ^b^Determined by GC and NMR.

Methyl 1-methylpyrrole-2-carboxylate (**25**) also reacts with 4-chlorobenzonitrile (**11**) to give **26** in good yields with catalysts **2**, **8** and **9** (Table 3). No significant decarboxylation of the pyrrole derivative was observed in the course of this reaction.

**Table 3:** Direct arylation of methyl 1-methylpyrrole-2-carboxylate (**25**) with 4-chlorobenzonitrile (**11**).^a^

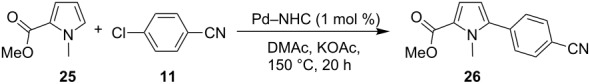			

Entry	Pd–NHC	Conv. (%)^b^	Yield (%)^b^

1	**1**	52	32
2	**2**	94	**78**
3	**4**	58	27
4	**5**	69	51
5	**6**	66	47
6	**7**	61	49
7	**8**	98	**83**
8	**9**	97	**81**

^a^Reaction conditions: Pd–NHC (0.01 mmol), 4-chlorobenzonitrile (**11**, 1 mmol), methyl 1-methylpyrrole-2-carboxylate (**25**, 2 mmol), KOAc (2 mmol), DMAc (3 mL), 20 h, 150 °C. ^b^Determined by GC and NMR.

Three aryl chlorides have also been coupled with 1-methylpyrrole (**27**, Table 4). A large excess of 1-methylpyrrole (**27**) was employed (4 equiv) in order to avoid the formation of 2,5-diarylated pyrroles. From 2- and 4-chlorobenzonitrile, **28** and **29** were obtained in high yields in the presence of complexes **8** and **9**. On the other hand, the formation of several side-products was observed during the coupling of 4-(trifluoromethyl)chlorobenzene (**13**) with this pyrrole derivative, and **30** was obtained in low yields (Table 4, entries 11–15).

**Table 4:** Direct arylation of 1-methylpyrrole (**27**) with chlorobenzene derivatives.^a^

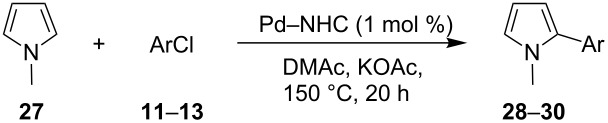					

Entry	ArCl	Pd–NHC	Product	Conv. (%)^b^	Yield (%)^b^

1 2 3 4 5 6	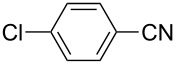 **11**	**1** **4** **6** **7** **8** **9**	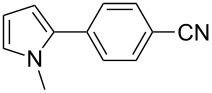 **28**	56 75 44 68 94 96	50 70 39 63 **83** **90**

7 8 9 10	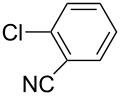 **12**	**1** **4** **8** **9**	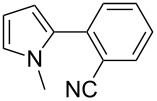 **29**	78 81 94 92	74 70 **88** **83**

11 12 13 14 15	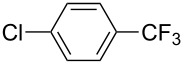 **13**	**2** **3** **4** **8** **9**	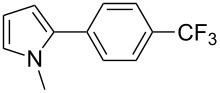 **30**	94 91 89 96 97	31 27 39 29 25

^a^Reaction conditions: Pd–NHC (0.01 mmol), aryl chloride (1 mmol), 1-methylpyrrole (**27**, 4 mmol), KOAc (2 mmol), DMAc (3 mL), 20 h, 150 °C. ^b^Determined by GC and NMR.

Finally, the reactivity of 1-phenylpyrrole (**31**) with two aryl chlorides was examined (Table 5). Again, good yields in **32** were obtained with complexes **2**, **8** and **9** for the coupling with 4-chlorobenzonitrile (**11**). 4-Chloroacetophenone (**15**) also gave **33** in good yields with complexes **8** and **9**.

**Table 5:** Direct arylation of 1-phenylpyrrole (**31**) with chlorobenzene derivatives.^a^

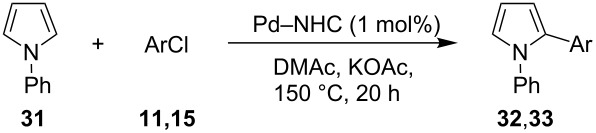					

Entry	ArCl	Pd–NHC	Product	Conv. (%)^b^	Yield (%)^b^

1 2 3 4 5 6 7 8	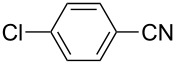 **11**	**1** **2** **4** **5** **6** **7** **8** **9**	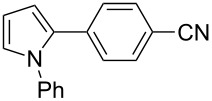 **32**	81 90 80 85 86 87 98 98	71 85 73 79 74 81 **89** **88**

9 10 11	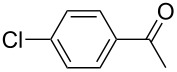 **15**	**2** **8** **9**	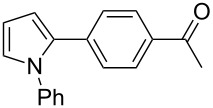 **33**	87 92 99	55 86 76

^a^Reaction conditions: Pd–NHC (0.01 mmol), aryl chloride (1 mmol), 1-phenylpyrrole (**31**, 4 mmol), KOAc (2 mmol), DMAc (3 mL), 20 h, 150 °C. ^b^Determined by GC and NMR.

## Conclusion

In summary, we have demonstrated that the regioselective C2 or C5 direct arylation of a range of pyrrole derivatives using electron-deficient aryl chlorides can be promoted by *N*-heterocyclic carbene ligands associated to palladium. So far, the reason for the influence of the nature of the carbene ligand on such couplings remains unclear. However, the presence of bulky N-substituents on the benzimidazole ring, such as 3,5-di-*tert*-butylbenzyl (**1**–**4**) or benzhydryl (**8**), appears to be favorable; whereas, 2-(2-ethoxy)phenoxyethyl substituent (**5**–**7**) generally led to lower yields. The presence of a 2-(2-ethyl)-1,3-dioxalane as N-substituent (**9**) was also found to be profitable. To our knowledge, these are the first examples of direct arylations of pyrroles by using aryl chlorides as the coupling partners and Pd-N-heterocyclic carbene complexes as the catalyst. Finally, as the major by-products are AcOK associated to HBr instead of metallic salts, this procedure is environmentally more attractive than the classical coupling procedures.

## Experimental

The reaction of benzimidazolium halide (2 equiv) with Pd(OAc)_2_ in DMSO according to Scheme 1 led to the formation of the desired complexes of Pd(II) in 53–87% yield. The crude product was recrystallized from a dichloromethane/diethyl ether mixture 1:3 at room temperature, which afforded the corresponding crystals. The new complexes were characterized by ^1^H NMR, ^13^C NMR, IR and elemental analysis techniques, which support the proposed structures.

As described in [49], the air and moisture-stable palladium-carbene complexes (**1–9**) were soluble in halogenated solvents and insoluble in nonpolar solvents. Palladium complexes exhibit a characteristic ν_(NCN)_ band typically at 1407–1477 cm^−1^. The formation of the Pd–NHC complexes was confirmed by the absence of the ^1^H NMR resonance signal of the acidic benzimidazolium C2–H. The ^13^C NMR spectra of Pd–NHC complexes exhibit a resonance signal in the 181.2–183.6 ppm range ascribed to the carbenic carbon atom, which is consistent with the reported values for Pd–NHC complexes [43]. NMR data showed that complexes **2** and **4**–**7** were *cis*/*trans* mixtures.

### General procedure for the preparation of the palladium–NHC complexes

As described in [50], to a solution of benzimidazolium salts (10 mmol) in DMSO (5 mL) was added palladium(II) diacetate (5 mmol) under argon, and the resulting mixture was stirred at room temperature for 2 h, then at 60 °C for 4 h, at 80 °C for 2 h and finally at 110 °C for 2 h. Volatiles were removed in vacuo, and the residue was washed twice with THF (5 mL). The complex was crystallized from dichloromethane/diethyl ether 1:3 at room temperature.

**Dibromo-bis[1-(3,5-di-*tert*-butylbenzyl)-3-(2-methoxyethyl)benzimidazol-2-ylidene]palladium(II) (1):** Yield: 0.29 g, 87%; mp 172–174 °C; ^1^H NMR (CDCl_3_, δ) 1.29 (t, *J* = 7.0 Hz, 4H, NCH_2_C*H*_2_OCH_3_), 1.31 (t, *J* = 7.0 Hz, 4H, NC*H*_2_CH_2_OCH_3_), 1.33 (s, 36H, NCH_2_C_6_H_3_(C(C*H*_3_)_3_)-3,5), 2.63 (s, 6H, NCH_2_CH_2_OC*H*_3_), 5.10 (s, 4H, NC*H*_2_C_6_H_3_(C(CH_3_)_3_-3,5)), 6.89–7.6 (m, 14H, NC_6_*H*_4_N and NCH_2_C_6_*H*_3_(C(CH_3_)_3_-3,5)); ^13^C NMR (CDCl_3_, δ) 31.5 (NCH_2_C_6_H_3_(C(*C*H_3_)_3_)-3,5), 34.8 (N*C*H_2_C_6_H_3_(C(CH_3_)_3_-3,5)), 35.0 (NCH_2_*C*H_2_OCH_3_), 41.0 (NCH_2_C_6_H_3_(*C*(CH_3_)_3_)-3,5)), 48.3 (NCH_2_CH_2_O*C*H_3_), 58.8 (N*C*H_2_C_6_H_3_(C(CH_3_)_3_-3,5)), 111.1, 111.2, 121.7, 122.3, 122.7, 122.9, 134.2, 134.6, 151.1, 151.3 (N*C*_6_H_4_N and NCH_2_*C*_6_H_3_(C(CH_3_)_3_-3,5)), 183.6 (Pd-*C_carbene_*); IR (cm^−1^) ν_(CN)_: 1407; Anal. calcd for C_50_H_68_N_4_PdBr_2_: C, 60.58; H, 6.91; N, 5.65; found: C, 60.47; H, 6.94; N, 5.63.

***cis/trans*-Dibromo-bis[1-(3,5-di-*tert*-butylbenzyl)-3-(3,4,5-trimethoxybenzyl)benzimidazol-2-ylidene]palladium(II) (2):** Yield: 0.29 g, 87%; mp 160–162 °C; ^1^H NMR (CDCl_3_, δ) 1.16, 1.21 (s, 36H, NCH_2_C_6_H_3_(C(C*H*_3_)_3_-3,5), 3.66, 3.80, 3.81, 3.86 (s, 18 H, NCH_2_C_6_H_2_(OC*H*_3_)_3_-3,4,5), 5.32, 5.37 (s, 4H, NC*H*_2_C_6_H_3_(C(CH_3_)_3_-3,5), 5.74, 5.79 (s, 4H, NC*H*_2_C_6_H_2_(OCH_3_)_3_-3,4,5), 6.09–7.39 (m, 18H, NC_6_*H*_4_N, NCH_2_C_6_*H*_3_(C(CH_3_)_3_-3,5 and NCH_2_C_6_*H*_2_(OCH_3_)_3_-3,4,5); ^13^C NMR (CDCl_3_, δ) 31.2, 31.3 (NCH_2_C_6_H_3_(C(*C*H_3_)_3_-3,5), 31.4, 31.7, 34.7, 34.8 (NCH_2_C_6_H_2_(O*C*H_3_)_3_-3,4,5), 41.0, 41.1 (NCH_2_C_6_H_3_(*C*(CH_3_)_3_-3,5), 53.2, 53.9 (N*C*H_2_C_6_H_3_(C(CH_3_)_3_-3, 5), 56.3, 56.4 (N*C*H_2_C_6_H_2_(OCH_3_)_3_-3,4,5), 104.4, 104.8, 111.8, 112.4, 121.1, 121.3, 123.4, 129.9, 130.4, 133.1, 133.5, 133.9, 134.3, 134.4, 134.7, 137.7, 151.2, 151.5, 153.5, 153.7 (N*C*_6_H_4_N, NCH_2_*C*_6_H_3_(C(CH_3_)_3_-3,5 and NCH_2_*C*_6_H_2_(OCH_3_)_3_-3,4,5), 181.2 and 182.3 (Pd-*C_carbene_*); IR (cm^−1^) ν_(CN)_: 1447; Anal. calcd for C_64_H_80_N_4_O_6_PdBr_2_: C, 60.64; H, 6.36; N, 4.42; found: C, 60.57; H, 6.54; N, 4.45.

**Dibromo-bis[1,3-bis(3,5-di-*tert*-butylbenzyl)benzimidazol-2-ylidene]palladium(II) (3):** Yield: 0.27 g, 82%; mp 248–250 °C; ^1^H NMR (CDCl_3_, δ) 1.18 (s, 72H, NCH_2_C_6_H_3_(C(C*H*_3_)_3_)-3,5), 5.80 (s, 8H, NC*H*_2_C_6_H_3_(C(CH_3_)_3_-3,5), 6.14–7.48 (m, 20H, NC_6_*H*_4_N and NCH_2_C_6_*H*_3_(C(CH_3_)_3_-3,5); ^13^C NMR (CDCl_3_, δ) 31.4 (NCH_2_C_6_H_3_(C(*C*H_3_)_3_)-3,5), 41.02 (NCH_2_C_6_H_3_(*C*(CH_3_)_3_)-3,5), 53.9 (N*C*H_2_C_6_H_3_(C(CH_3_)_3_-3,5), 111.6, 112.2, 121.3, 121.5, 122.3, 122.8, 133.4, 134.4, 134.6, 151.1, 151.2 (N*C*_6_H_4_N and NCH_2_*C*_6_H_3_(C(CH_3_)_3_-3,5)), 182.5 (Pd-*C_carbene_*); IR (cm^−1^) ν_(CN)_: 1477; Anal. calcd for C_74_H_100_N_4_PdBr_2_: C, 67.75; H, 7.68; N, 4.27; found: C, 67.72; H, 7.64; N, 4.27.

***cis/trans*-Dichloro-bis[1-(3,5-di-*tert*-butylbenzyl)-3-(2,3,4,5,6-pentamethylbenzyl)benzimidazol-2-ylidene]palladium(II) (4):** Yield: 0.27 g, 82%; mp 310–312 °C; ^1^H NMR (CDCl_3_, δ) 1.27, 1.29 (s, 36 H, NCH_2_C_6_H_3_(C(C*H*_3_)_3_-3,5)), 2.20, 2.23, 2.24, 2.29, 2.30, 2.34 (s, 30H, NCH_2_C_6_(C*H*_3_)_5_-2,3,4,5,6), 5.30 and 5.40 (s, 4H, NC*H*_2_C_6_(CH_3_)_5_-2,3,4,5,6), 5.53, 5.54 (s, 4H, NC*H*_2_C_6_H_3_(C(CH_3_)_3_-3,5)), 6.04–7.55 (m, 14H, NC_6_*H*_4_N and NCH_2_C_6_*H*_3_(C(CH_3_)_3_-3,5)); ^13^C NMR (CDCl_3_, δ) 31.3, 31.4 (NCH_2_C_6_H_3_(C(C*H*_3_)_3_-3,5), 41.0, 41.1 (NCH_2_C_6_H_3_(*C*(CH_3_)_3_-3,5)), 17.1, 17.2, 17.3, 17.6, 17.7, 17.8 (NCH_2_C_6_(*C*H_3_)_5_-2,3,4,5,6), 51.2, 51.3 (N*C*H_2_C_6_(CH_3_)_5_-2,3,4,5,6), 51.5, 51.6 (N*C*H_2_C_6_H_3_(C(CH_3_)_3_-3,5)), 111.2, 111.4, 111.8, 121.3, 121.5, 122.0, 122.5, 122.7, 122.8, 128.5, 128.6, 132.9, 133.0, 134.3, 134.4, 134.5, 134.6, 134.8, 134.9, 135.1, 151.0, 151.1 (N*C*_6_H_4_N and NCH_2_*C*_6_H_3_(C(CH_3_)_3_-3,5)), 182.4, 182.5 (Pd-*C_carbene_*); IR (cm^−1^) ν_(CN)_: 1451; Anal. calcd for C_68_H_84_N_4_PdCl_2_: C, 71.97; H, 7.46; N, 4.94; found: C, 71.92; H, 7.64; N, 4.97.

***cis/trans*-Dibromo-bis[1-(2,4,6-trimethylbenzyl)-3-(2-(2-ethoxy)phenoxyethyl)benzimidazol-2-ylidene]palladium(II) (5):** Yield: 0.33 g; 81%; mp 238–240 °C; ^1^H NMR (CDCl_3_, δ) 1.23, 1.39 (t, *J* = 7.0 Hz, 6H, NCH_2_CH_2_OC_6_H_4_(OCH_2_C*H*_3_)-2), 2.29, 2.34, 2.35, 2.36 (s, 18H, NCH_2_C_6_H_2_(C*H*_3_)-2,4,6), 3.89, 4.01 (q, *J* = 7.0 Hz, 4H, NCH_2_CH_2_OC_6_H_4_(OC*H*_2_CH_3_)-2), 4.81, 4.83 (t, *J* = 5.9 Hz, 4H, NCH_2_C*H*_2_OC_6_H_4_(OCH_2_CH_3_)-2), 5.39, 5.41 (t, *J* = 5.9, 4H, NC*H*_2_CH_2_OC_6_H_4_(OCH_2_CH_3_)-2), 6.03, 6.13 (s, 4H, NC*H*_2_C_6_H_2_(CH_3_)-2,4,6), 6.80–7.77 (m, 20H, NC_6_*H*_4_N, NCH_2_CH_2_OC_6_*H*_4_(OCH_2_CH_3_)-2, NCH_2_C_6_*H*_2_(CH_3_)-2,4,6); ^13^C NMR (CDCl_3_, δ) 15.1, 15.3 (NCH_2_CH_2_OC_6_H_4_(OCH_2_*C*H_3_)-2), 21.0, 21.1, 21.2, 21.3 (NCH_2_C_6_H_2_(*C*H_3_)-2,4,6), 48.0, 48.1 (NCH_2_CH_2_OC_6_H_4_(O*C*H_2_CH_3_)-2), 50.1, 50.6 (NCH_2_*C*H_2_OC_6_H_4_(OCH_2_CH_3_)-2), 64.0, 64.1 (N*C*H_2_CH_2_OC_6_H_4_(OCH_2_CH_3_)-2), 67.8, 67.9 (N*C*H_2_C_6_H_2_(CH_3_)-2,4,6), 111.3, 111.5, 112.8, 113.3, 120.7, 120.9, 121.4, 122.9, 128.0, 129.4, 129.6, 134.6, 135.7, 138.4, 138.6, 138.9, 148.0, 148.5, 148.6 (N*C*_6_H_4_NNCH_2_CH_2_O*C*_6_H_4_(OCH_2_CH_3_)-2, NCH_2_*C*_6_H_2_(CH_3_)-2,4,6), 182.2, 182.3 (Pd-*C_carbene_*); IR (cm^−1^) ν_(CN)_: 1448; Anal. calcd for C_54_H_60_N_4_O_4_PdBr_2_: C, 59.21; H, 5.52; N, 5.12; found: C, 59.27; H, 5.54; N, 5.13.

***cis/trans*-Dichloro-bis[1-(2-(2-ethoxy)phenoxyethyl)-3-(4-methylbenzyl)benzimidazol-2-ylidene] palladium(II) (6):** Yield: 0.32 g, 66%; mp 235–237 °C; ^1^H NMR (CDCl_3_, δ) 1.43, 1.45 (t, *J* = 6.9 Hz, 6H, NCH_2_CH_2_OC_6_H_4_(OCH_2_C*H*_3_)-2), 2.29, 2.35 (s, 6H, NCH_2_C_6_H_4_(C*H*_3_)-4), 3.98, 4.03 (q, *J* = 7.0 Hz, 4H, NCH_2_CH_2_OC_6_H_4_(OC*H*_2_CH_3_)-2), 4.57, 4.82 (t, *J* = 5.0 Hz, 4H, NCH_2_C*H*_2_OC_6_H_4_(OCH_2_CH_3_)-2), 5.27, 5.42 (t, *J* = 5.0 Hz, 4H, NC*H*_2_CH_2_OC_6_H_4_(OCH_2_CH_3_)-2), 5.97, 6.15 (s, 4H, NC*H*_2_C_6_H_4_(CH_3_)-4), 6.68–8.56 (m, 24H, NC_6_*H*_4_N, NCH_2_CH_2_OC_6_*H*_4_(OCH_2_CH_3_)-2, NCH_2_C_6_*H*_4_(CH_3_)-4); ^13^C NMR (CDCl_3_, δ) 15.0, 15.1 (NCH_2_CH_2_OC_6_H_4_(OCH_2_*C*H_3_)-2), 21.1, 21.2 (NCH_2_C_6_H_4_(*C*H_3_)-4), 48.1, 48.3 (NCH_2_CH_2_OC_6_H_4_(O*C*H_2_CH_3_)-2), 52.1, 52.2 (NCH_2_*C*H_2_OC_6_H_4_(OCH_2_CH_3_)-2), 64.0, 64.1 (N*C*H_2_CH_2_OC_6_H_4_(OCH_2_CH_3_)-2), 68.3, 68.6 (N*C*H_2_C_6_H_4_(CH_3_)-4), 110.8, 111.1, 112.2, 112.8, 113.1, 120.7, 120.9, 121.2, 123.0, 123.1, 127.6, 127.7, 127.8, 129.3, 129.5, 132.6, 134.1, 135.6, 137.4, 137.6, 148.0, 148.4, 148.6 (N*C*_6_H_4_NNCH_2_CH_2_O*C*_6_H_4_(OCH_2_CH_3_)-2, NCH_2_*C*_6_H_4_(CH_3_)-4), 182.0, 182.1 (Pd-*C_carbene_*); IR (cm^−1^) ν_(CN)_: 1407; Anal. calcd for C_50_H_52_N_4_O_4_PdCl_2_: C, 63.19; H, 5.52; N, 5.90; found: C, 63.18; H, 5.50; N, 5.93.

***cis/trans*-Dichloro-bis[1-(2-(2-ethoxy)phenoxyethyl)-3-(3-methoxybenzyl)benzimidazo-2-ylidene]palladium(II) (7):** Yield: 0.22 g, 53%; mp 205–207 °C; ^1^H NMR (CDCl_3_, δ) 1.43, 1.45 (t, *J* = 7.0 Hz, 6H, NCH_2_CH_2_OC_6_H_4_(OCH_2_C*H*_3_)-2), 3.66, 3.75 (s, 6H, NCH_2_C_6_H_4_(OC*H*_3_)-3), 3.97, 4.03 (q, *J* = 7.0 Hz, 4H, NCH_2_CH_2_OC_6_H_4_(OC*H*_2_CH_3_)-2)), 4.60, 4.83 (t, *J* = 5.6 Hz, 4H, NCH_2_C*H*_2_OC_6_H_4_(OCH_2_CH_3_)-2), 5.29, 5.43 (t, *J* = 5.7 Hz, 4H, NC*H*_2_CH_2_OC_6_H_4_(OCH_2_CH_3_)-2), 6.01, 6.18 (s, 4H, NC*H*_2_C_6_H_4_(OCH_3_)-3), 6.67–7.86 (m, 24H, NC_6_*H*_4_N, NCH_2_CH_2_OC_6_*H*_4_(OCH_2_CH_3_)-2, NCH_2_C_6_*H*_4_(OCH_3_)-3); ^13^C NMR (CDCl_3_, δ) 15.0, 15.3 (NCH_2_CH_2_OC_6_H_4_(OCH_2_*C*H_3_)-2), 48.0, 48.3 (NCH_2_CH_2_OC_6_H_4_(O*C*H_2_CH_3_)-2), 52.3, 52.4 (NCH_2_C_6_H_4_(O*C*H_3_)-3), 55.5, 55.7 (NCH_2_*C*H_2_OC_6_H_4_(OCH_2_CH_3_)-2), 63.9, 64.0 (N*C*H_2_CH_2_OC_6_H_4_(OCH_2_CH_3_)-2), 68.3, 68.6 (N*C*H_2_C_6_H_4_(OCH_3_)-3), 112.3, 113.1, 114.7, 120.0, 120.7, 120.9, 123.1, 123.2, 129.8, 134.0, 134.1, 135.6, 137.0, 137.3, 148.0, 148.4, 160.0, 160.3 (N*C*_6_H_4_N NCH_2_CH_2_O*C*_6_H_4_(OCH_2_CH_3_)-2, NCH_2_*C*_6_H_4_(OCH_3_)-3), 182.0, 182.2 (Pd-*C_carbene_*); IR (cm^−1^) ν_(CN)_: 1444; Anal. calcd for C_50_H_52_N_4_O_6_PdCl_2_: C, 61.14; H, 5.34; N, 5.70; found: C, 61.21; H, 5.37; N, 5.73.

**Dibromo-bis[1-(3-methylbenzyl)-3-(benzhydryl)]benzimidazol-2-ylidene]palladium(II) (8):** Yield: 0.35 g, 60%; mp 230–232 °C; ^1^H NMR (CDCl_3_, δ) 2.12 (3-C*H*_3_C_6_H_5_), 5.72 (s, 2H, (3-CH_3_)(C_6_H_5_)-C*H*_2_), 6.74–7.79 (m, 19H, C*H*(C_6_*H*_5_)_2_, C_6_*H*_4_ and 3-CH_3_C_6_*H*_5_); ^13^C NMR (CDCl_3_, δ) 21.3 (3-(*C*H_3_)(C_6_H_5_)), 52.0 (3-(CH_3_)(C_6_H_5_)-*C*H_2_), 67.5 (*C*H(C_6_H_5_), 112.4, 123.6, 125.5, 128.6, 128.7, 128.9, 129.1, 133.4, 133.8, 134.9, 135.9, 136.1, 137.6, 138.0, 138.2, 138.4 (3-(CH_3_)(*C*_6_H_5_), CH(*C*_6_H_5_) and *C*_6_H_4_), 183.5 (Pd-*C_carbene_*); IR (cm^−1^) ν_(CN)_: 1412; Anal. calcd for C_56_H_46_N_4_Br_2_Pd: C, 64.60; H, 4.45; N, 5.38; found: C, 64.58; H, 4.49; N, 5.46.

**Dibromo-bis[1-(benzyl)-3-(2-(2-ethyl)-1,3-dioxalane)]benzimidazol-2-ylidene]palladium(II) (9):** Yield: 0.31 g, 62%; mp 282–284 °C; ^1^H NMR (CDCl_3_, δ) 2.27 (m, 2H, NCH_2_C*H*_2_CH), 3.83 and 3.99 (t, 4H, *J* = 6.6 Hz, NCH_2_CH_2_CHO_2_C*H*_2_C*H*_2_), 5.01 (m, 3H, NC*H*_2_CH_2_CH and NCH_2_CH_2_C*H*), 6.01 (s, 2H, (C_6_H_5_)-C*H*_2_), 7.0–7.92 (m, 9H, (C_6_*H*_5_)CH_2_ and C_6_*H*_4_); ^13^C NMR (CDCl_3_, δ) 33.7 (NCH_2_*C*H_2_CH), 40.8 (N*C*H_2_CH_2_CH), 64.9 (NCH_2_CH_2_CHO_2_*C*H_2_*C*H_2_), 101.6 (NCH_2_CH_2_*C*HO_2_CH_2_CH_2_), 101.9, 111.3, 112.2, 123.7, 128.3, 128.5, 128.8, 128.9, 133.9, 134.4, 136.6 (*C*_6_H_5_CH_2_ and *C*_6_H_4_), 181.7 (Pd-*C_carbene_*); IR (cm^−1^) ν_(CN)_: 1408; Anal. calcd for C_38_H_40_O_4_N_4_PdBr_2_: C, 51.69; H, 4.57; N, 6.35; found: C, 51.60; H, 4.61; N, 6.37.

### General Procedure for direct arylations

As described in [47], in a typical experiment, the aryl chloride (1 mmol), heteroaryl derivative (2 or 4 mmol) (see Table 1–5) and KOAc (2 mmol) were introduced in a Schlenk tube, equipped with a magnetic stirring bar. The Pd complex (0.01 mmol, see Table 1–5) and DMAc (3 mL) were added, and the Schlenk tube was purged several times with argon. The Schlenk tube was placed in a preheated oil bath at 150 °C, and the reaction mixture was stirred for 20 h. Then, the reaction mixture was analysed by gas chromatography to determine the conversion of the aryl chloride. The solvent was removed by heating of the reaction vessel under vacuum and the residue was charged directly onto a silica-gel column. The products were eluted by using an appropriate ratio of diethyl ether and pentane.
